# Longitudinal development and clinical predictors of financial toxicity among radiation oncology patients: final results of the SOCOFIN study

**DOI:** 10.1007/s00066-025-02479-9

**Published:** 2025-10-23

**Authors:** Anna Luisa Kreuser, Sonia Ziegler, Stephanie Bendrich, Alexander Ziegler, Thomas Asendorf, Oliver Rick, Leif Hendrik Dröge, Martin Leu, Manuel Guhlich, Jan Oelmann, Laura Anna Fischer, Jann Fischer, Friederike Braulke, Stefan Rieken, Rami El Shafie

**Affiliations:** 1 Department of Radiation Oncology, University Medical Center Göttingen, Göttingen, Germany; 2 Department of Medical Statistics, University Medical Center Göttingen, Göttingen, Germany; 3 Göttingen—Comprehensive Cancer Center Göttingen, University Medical Center Göttingen, Göttingen, Germany; 4 Department of Radiation Oncology, Medical University of Lausitz—Carl Thiem, Cottbus, Germany; 5 Oncological Rehabilitation, Clinic of Reinhardshöhe, Bad Wildungen, Germany

**Keywords:** Financial burden, Cancer survivorship, Supportive care, Radiation therapy

## Abstract

**Purpose:**

Financial toxicity (FT) associated with cancer and its treatment has become increasingly important. This study investigated factors associated with the development of FT during radiation therapy (RT). SOCOFIN was the first longitudinal prospective study to systematically evaluate FT in the context of RT.

**Methods:**

Financial toxicity was measured with the Comprehensive Score for Financial Toxicity (COST-12) at RT initiation, completion, and at 3 months afterwards. Secondary endpoints included socioeconomic factors, health-related quality of life (EORTC QLQ-C30), depression (PHQ-9), coping mechanisms, and sense of coherence. The data were collected digitally; missing data were estimated using multiple imputation with chained equations.

**Results:**

Between July 2023 and June 2024, 230 patients were recruited. Analyses were performed on 170 records. During RT, FT did not increase; a slight overall decrease was descriptively observed. Of seven tumor groups, the highest difference in FT at baseline was measured between prostate (median 33) and pelvic cancer patients (median 19), reaching statistical significance (Kruskal–Wallis test, *p* = 0.01). Nonetheless, tumor entity was not found to be a significant predictor of FT following RT in multivariate linear regression models. While factors associated with FT differed between timepoints, financial difficulties at baseline predicted the occurrence of FT most strongly (*p* < 10^−13^) and persistently.

**Conclusion:**

Predictors of FT were predominantly socioeconomic, such as baseline financial difficulties, net income, employment stability, and sense of coherence, which superseded tumor- or treatment-specific variables. The findings of this study underscore the necessity of multifactorial, early screening before RT to mitigate FT among radiation oncology patients.

## Introduction

Financial burden associated with cancer treatment has emerged as a meaningful concern for patients and may encompass direct and indirect costs leading to objective financial stress. In combination with subjective financial stress and insufficient coping mechanisms, this could lead to “financial toxicity” (FT) [1]. Research has demonstrated that FT has effects on quality of life and is associated with elevated mortality rates [2, 3]. In the United States, where healthcare expenditures are predominantly privatized, FT has been linked to elevated mortality (HR = 1.79), particularly among younger and female patients [3]. It is noteworthy that FT could be detected in patients both with and without health insurance [4]. Financial toxicity is increasingly recognized as an essential aspect of cancer care, significantly impacting patients’ quality of life, adherence to treatment, and overall health outcomes [1]. Existing literature on specific tumor groups that are particularly susceptible to FT is limited. However, there is evidence from studies in breast and head and neck (SCCHN) cancer patients that suggests a higher risk of experiencing FT [5, 6].

In nations that have implemented universal healthcare systems, such as Germany, Canada, and the United Kingdom, the concept of FT is gaining recognition. Research indicates that income loss, rather than copayments, is the predominant factor contributing to FT in these contexts [7, 8]. In a German study, approximately 37% of advanced cancer patients encountered a reduction in income, and FT was associated with diminished quality of life, depression, and anxiety [7]. Depression and anxiety among thyroid cancer patients were negatively correlated with a higher sense of coherence, which is an indicator of resilience and coping ability [9]. Other cross-sectional studies found that 33 to 41% of patients receiving radiation therapy (RT) in Germany experienced financial distress, while the risk factors of low net income, income loss, and direct costs were associated with a higher probability of FT [10, 11].

A multitude of factors have been identified as risk indicators for the development of FT, including but not limited to low income, financial hardship at baseline [12], changes in employment status, younger age, advanced tumor stage, and systemic therapies [13]. Patients undergoing RT might be particularly vulnerable to FT due to the considerable time burden on working-age patients, which can lead to job and income loss or other economic hardships [5].

Given the discussed differences between healthcare systems, both international and German studies underscore the multifactorial nature of FT, emphasizing its association with socioeconomic conditions, healthcare structure, and psychosocial coping resources. Despite growing awareness, the trajectory and predictors of FT in radiation oncology settings remain poorly defined. Identifying patients at risk for FT at an early stage of treatment could enable healthcare providers to implement proactive interventions, ultimately improving both clinical and patient-centered outcomes. This first-time longitudinal study of FT in Germany under the name SOCOFIN (Efficacy of a Structured Social Consultation and Support in Reducing the Financial Burden of Radiotherapy) investigates pretreatment clinical and sociodemographic predictors among radiation oncology patients.

## Methods

### Ethics

The institutional review board granted ethical approval for the study application (entry number 18/3/23). The acquisition of written informed consent preceded the initiation of any trial-specific questionnaires. The SOCOFIN study was conducted in accordance with the Good Clinical Practice (GCP) guidelines. In accordance with these guidelines, the study has been registered on a public, internationally recognized study registry (clinicaltrials.gov) and can be found under the identification number NCT06278831. Moreover, the study (ARO 2024-11) was endorsed by the Radiation Oncology Working Group (ARO) of the German Cancer Society (DKG).

### Study design and data collection

A total of 230 cancer patients undergoing radiation therapy at a certified German tertiary cancer center between July 2023 and June 2024 were enrolled. Inclusion criteria entailed a minimum age of 18 years, a confirmed cancer diagnosis, scheduled radiation therapy, life expectancy of at least 3 months, Karnofsky performance score (KPS) of at least 70%, and access to the internet for digital questionnaires. The data were collected digitally using an electronic case report form based on the REDCap (Research Electronic Data Capture) survey platform (Vanderbilt University, Nashville, TN, USA) as well as a mobile application, Patienta (© 2024 ACALTA GmbH).

### Measurements

The primary outcome of FT was assessed using the Comprehensive Score for Financial Toxicity (COST-FACIT) [14] at three timepoints: baseline (t0), after RT (t1), and at a 3-month follow-up (t2). With smaller values indicating more FT, the severity of FT was divided into grades ranging from none to mild, moderate, and severe [15]. Secondary variables included sociodemographic factors; clinical data (tumor diagnosis and treatment, tumor stage, KPS, Charlson Comorbidity Index [CCI]); and psychosocial factors such as depression (Patient Health Questionnaire, PHQ-9), quality of life (EORTC QLQ C30), coping strategies, sense of coherence (sense of coherence score), and social support (social network index).

### Statistical analyses

The analyses were executed using Python version 3.12 in conjunction with the integrated development environment PyCharm version 2024.2.1 and R with RStudio version 2023.12.0 + 369 (R Foundation, Vienna, Austria). All graphics were created using Python 3.12. The descriptive analyses were conducted on 170 patients without imputations after incomplete data had been filtered out. After exclusions due to loss to follow-up, comprehensive inferential statistical analyses were conducted on 161 patients. Missing data were handled by multiple imputations with chained equations. The highest proportion of longitudinal nonresponse was 7.4% after RT. Longitudinal changes were evaluated using repeated-measures Friedman tests, while nominal data differences, such as between tumor groups or genders, were assessed using Kruskal–Wallis or Mann–Whitney U tests. Post-hoc Dunn tests were calculated with Bonferroni corrections. Spearman correlation coefficients were calculated for ordinal data and higher data scales. Multivariate linear regression models including the most important secondary outcomes were conducted for each timepoint to identify significant predictors of FT while controlling for multiple potential confounders. Moreover, separate multivariate linear regression models incorporating the most relevant treatment-associated parameters were calculated for each timepoint. The selection of confounders for the multivariate regression models was based on logical considerations and a maximum level of 20 degrees of freedom before fitting the respective models using the Akaike information criterion (AIC). Alpha levels were set to below 0.05, with the exception of the calculations for the Kruskal–Wallis tests on seven subgroups of tumor diagnoses, in order to maintain adequate test power (α = 0.1). The 95% confidence intervals (CI) are given.

## Results

**Fig. 1:**
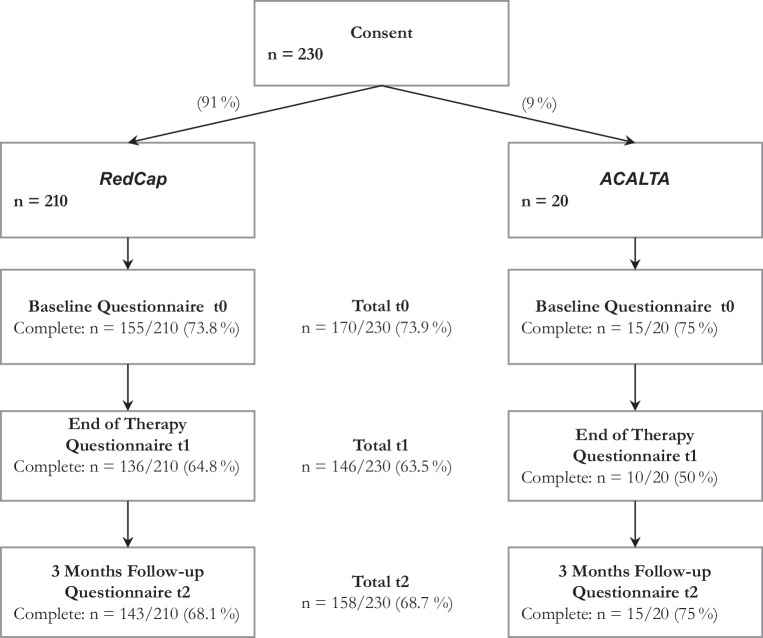
Patient recruitment and follow-ups

In summary, 74% of the patients who were recruited completed all of the questionnaires at baseline, as depicted by the flowchart in Fig. 1. A total of 161 participants (70%) remained in the study until the follow-up surveys by completing the minimum requirement of filling out all questionnaires at least twice.

### Study population (*n* = 170)

Table 1 presents the main characteristics of the study population before RT. The median patient age was 64 years (IQR 15) and 56.5% were female. The most common diagnosis was breast cancer (31.8%), followed by prostate cancer (15.9%). Approximately half of the patients had advanced tumors (UICC stages III or IV), and one third received palliative radiation therapy. The median KPS was 90%, and most (85%) were covered by statutory health insurance as opposed to private health insurance. Systemic therapy was administered to 58% of patients. While 20% of patients were hospitalized during RT, 19% reported hospitalization within 3 months following RT completion. Overall, 60% reported commuting distances between 25 and 50 km or more to the treatment center. The median household income was € 2500–3500 per month with two earners per household.

**Table 1:** Patient characteristics at baseline

	Absolute number	Proportion in %	Statistics
*Age (years)*			
Mean (SD)	–	–	62 (12.4)
Median (Q1–Q3)	–	–	64 (70–55)
*Gender*			
Female	96	56.5	–
Male	74	43.5	–
*Tumor diagnosis*			
Breast cancer	54	31.8	–
Prostate cancer	27	15.9	–
Gastrointestinal cancer	21	12.4	–
Lung cancer	17	10.0	–
Pelvic cancer (gynecological or urological)	11	6.5	–
Head and neck cancer	9	5.3	–
Primary brain tumor	6	3.5	–
Other	25	14.7	–
*Charlson Comorbidity Index*			
Mean (SD)	–	–	5.7 (2.7)
Median (Q1–Q3)	–	–	5 (4–7)
*KPS (%)*			
Mean (SD)	–	–	90.8 (9.5)
Median (Q1–Q3)	–	–	90 (90–100)
*Prior radiation therapy in the last 12 months*			
No	159	95.8	–
Yes	7	4.2	–
*Number of planned radiation fractions*			
Mean (SD)	–	–	20.7 (8.8)
Median (Q1–Q3)	–	–	20 (13–30)
*UICC tumor stage*			
Extensive (III or IV)	84	49.4	–
Limited (I or II)	60	35.3	–
Not applicable	26	15.3	–
*Systemic therapy*			
Chemotherapy, immunotherapy, targeted therapy	98	57.6	–
None, hormone therapy, other	72	42.4	–
*Therapy mode*			
Curative	113	67.7	–
Palliative	54	32.3	–
*Distance to treatment center*			
< 5 km	20	12.4	–
5–10 km	14	8.7	–
10–25 km	30	18.9	–
25–50 km	71	43.9	–
> 50 km	26	16.1	–
*Monthly net income per household*			
< € 1250	16	9.4%	–
€ 1250 to € 2500	47	27.6%	–
€ 2500 to € 3500	38	22.4%	–
€ 3500 to € 5000	34	20%	–
> € 5000	16	9.4%	–
Prefer not to answer	19	11.2%	–
*Health insurance*			
Public	143	74.1	–
Private	27	15.7	–

*SD* Standard Deviation, *Q1* first quartile, *Q3* third quartile, *KPS* Karnofsky Performance Score

### Longitudinal development of FT (*n* = 161)

**Fig. 2:**
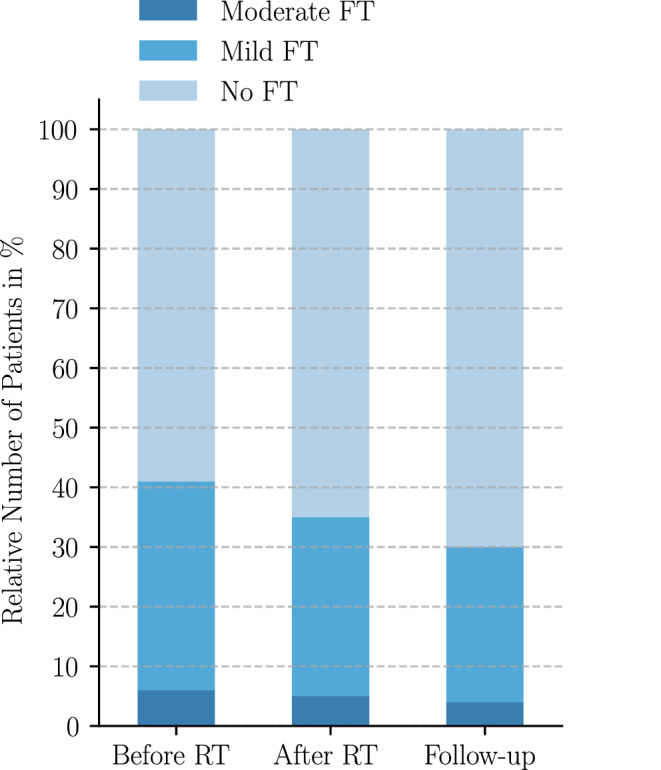
Severity of financial toxicity (*FT*) over time measured by the COST score (follow-up meaning 3 months after radiation therapy (RT))

The proportion of patients who experienced FT decreased from baseline (40.4%) to after RT (34.8%) and further until the 3‑month follow-up (30.4%), as shown in Fig. 2 and Appendix Table 2.

**Fig. 3:**
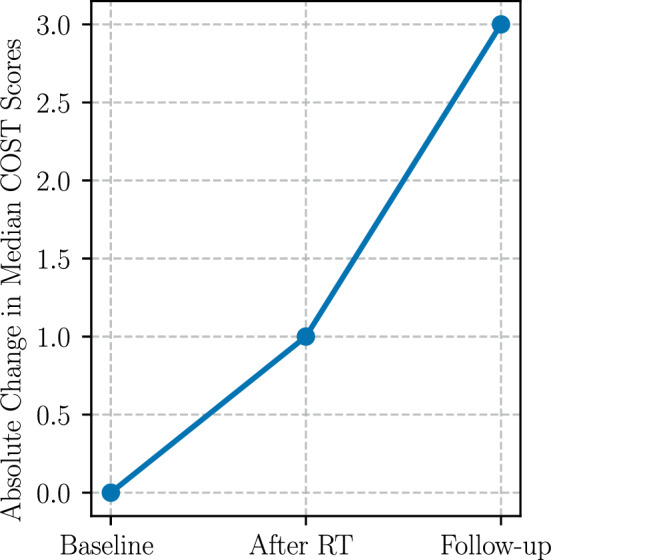
Absolute change in median COST scores over time (follow-up meaning 3 months after RT). Higher COST scores indicate lower financial toxicity

Overall, median COST scores showed modest improvement over the study period (baseline: 29; post-RT: 30; follow-up: 32), as shown in Fig. 3. Repeated-measures Friedman tests showed statistically significant differences in COST scores (Appendix Table 3) over the three timepoints (*p* = 0.017); however, no post-hoc test could decipher a significant difference between only two timepoints (Appendix Table 4).

Furthermore, repeated-measures Friedman tests of the COST scores over time could not find significant longitudinal differences between subgroups according to tumor diagnosis (*p* = 0.16–0.49). In contrast, repeated-measures Friedman tests of the COST scores over time across FT severity groups revealed different patterns: a significant change in COST scores over time was found in the subgroup of mild FT over all three timepoints (*p* = 0.02), as shown in Appendix Table 5. Nevertheless, post-hoc tests again showed no significant difference between only two timepoints (Appendix Table 6). Descriptive analyses reveal an improvement in FT in the subgroup with mild FT at baseline over time, with a median COST score of 21 at baseline and increasing to 22 after RT and to 24 points 3 months thereafter (Appendix Fig. 7).

Moreover, subgroup analyses of patients categorized by monthly net income per household revealed a statistically significant change in the COST score over time among patients with incomes ranging from 2500 to 3500 euros. Median COST scores in this patient group exhibited an increase from a median of 31 to 32 points over the course of the study (Appendix Fig. 8).

**Fig. 4:**
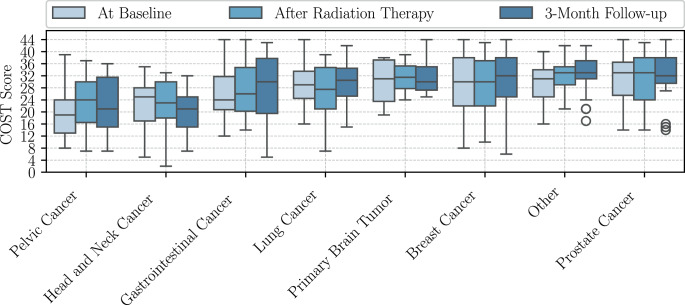
Distribution of COST scores among tumor diagnosis groups over time. Pelvic cancer includes cervical, uterine, endometrial, renal pelvis, urinary bladder, and renal cell cancer. Other cancer includes multiple myeloma, sarcoma (e.g., pleomorphic sarcoma, leiomyosarcoma, myxoid liposarcoma, malignant peripheral nerve sheath tumor), non-Hodgkin’s lymphoma (e.g., diffuse large B‑cell lymphoma, anaplastic large cell lymphoma, B‑cell acute lymphoblastic leukemia, marginal zone lymphoma of the orbit), squamous cell carcinoma, malignant melanoma, malignant thymoma, neuroendocrine breast cancer, anaplastic thyroid carcinoma

Differences in the COST scores between tumor groups were explored with Kruskal–Wallis tests (Table 7) and yielded highly significant differences at all timepoints, with the highest significance at the 3‑month follow-up (*p* = 0.009). As illustrated in Fig. 4, patients diagnosed with pelvic tumors exhibited the highest FT prior to RT (median 19). However, following RT, patients with head and neck cancer demonstrated the highest FT (median 23) and at 3 months after RT even worse FT (median 21).

Post-hoc Dunn–Bonferroni tests proved that patients with pelvic tumors had significantly higher FT than patients with breast (*p* = 0.04) or prostate cancer (*p* = 0.01) at baseline (Appendix Table 8). There was no significant difference detected between tumor groups after RT. However, at the 3‑month follow-up (Appendix Table 9), SCCHN patients had higher FT than breast (*p* = 0.06) and prostate cancer patients (*p* = 0.04).

Mann–Whitney U tests (Appendix Table 10) were performed to evaluate differences in COST scores among subgroups defined by secondary outcomes. Regarding hospitalization during and following RT, no significant differences in COST scores were observed at any timepoint. In contrast, systemic therapy was significantly associated with financial toxicity: patients who received chemotherapy, targeted therapy, or immunotherapy consistently reported lower COST scores, indicating higher financial toxicity (*p* = 0.02–0.04) compared to those who received no systemic therapy or only hormonal therapy. There were no statistically significant results for gender at any timepoint.

#### Univariate associations with FT

A multitude of variables exhibited substantial correlations with COST scores across all designated timepoints (Appendix Table 11). Higher levels of QLQ financial difficulties at baseline were most strongly associated with higher levels of greater FT at baseline, after RT, and at follow-up. Conversely, lower household income and income loss following RT have been demonstrated to be associated with lower COST scores, suggesting a heightened financial burden. The present study found a positive correlation between higher age, KPS, sense of coherence, and improved FT. Additionally, a weak but significant association was identified between fatigue and increased FT. No correlation was observed between UICC tumor stages and FT at baseline; however, after radiation and at the 3‑month follow-up, a significant correlation was revealed. Distances to the radiation center did not demonstrate a significant correlation with COST scores at any of the timepoints.

#### Coping mechanisms for FT

The most frequently reported coping strategy for financial burden was lifestyle modification (27% after RT and 35% 3 months thereafter), followed by use of passive financial resources (22 and 25%, respectively), as depicted in Appendix Table 12. It is noteworthy that all available coping mechanisms were selected with increasing frequency at the 3‑month follow-up, with the most significant rise observed in active financial spending, which includes the acquisition of loans (1.2 after RT and 2.4% 3 months thereafter).

### Predictors of financial toxicity

#### Multivariate regression models with secondary outcomes

Baseline financial difficulties consistently emerged as the strongest predictor of FT across all timepoints, with extremely small *p*-values (*p* < 10^−8^). Other significant predictors of FT included net income at baseline, changes in employment status, KPS at baseline, and sense of coherence at baseline (*p* = 0.034).

The regression model for the COST score at baseline illustrates the significance of socioeconomic factors as well as of the KPS (*p* = 0.002), which had a significant impact on FT at baseline (Appendix Table 13). The most important predictors were QLQ financial difficulties (*p* = 1.45 $\cdot$ 10^−8^) and monthly net income (*p* = 2.43 $\cdot$ 10^−4^). A worse employment situation (*p* = 0.002) and sense of coherence (*p* = 0.034) also contributed to higher FT. Number of persons per household, QLQ pain, QLQ dyspnea, change of income, improvement of occupational situation, employment at baseline, health insurance, physical labor, and social network showed no significant impact on the primary outcome.

**Fig. 5:**
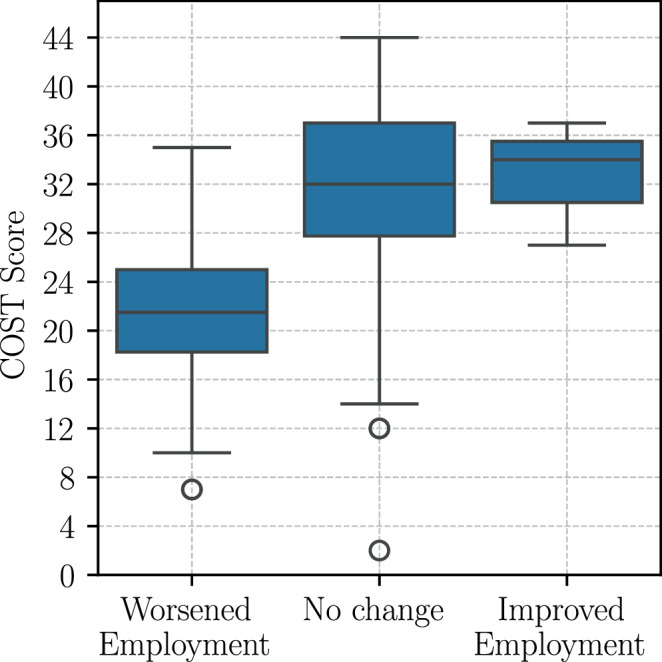
COST score after radiation therapy by change in employment situation after radiation therapy

The multivariate linear regression model for FT after RT found the most significant predictors to be, once again, QLQ financial difficulties (*p*-value < 10^−11^), as presented in Appendix Table 14. Other socioeconomic factors, like no change in employment status (*p* = 0.004 and 0.01) and no changes in income following radiation treatment (*p* = 0.047) were shown to significantly influence FT after RT: Patients who maintain a stable employment status after radiation treatment, in contrast to those who experience a deterioration, were predicted to demonstrate an average increase of 9.58 points (95% CI 2.32–16.84) in COST scores (*p* = 0.011), i.e., to experience less FT. In this study, as demonstrated in Fig. 5, the median COST scores of patients who experienced a deterioration in their employment status were 10 points lower (median 22) than those of patients who maintained stability in their employment (median 32). The subsequent predictors, which included net income, academic education, household size, fatigue, nausea, social support, gender, insurance type, employment status at baseline, and tumor stage were not found to be significant for the primary outcome after RT.

**Fig. 6:**
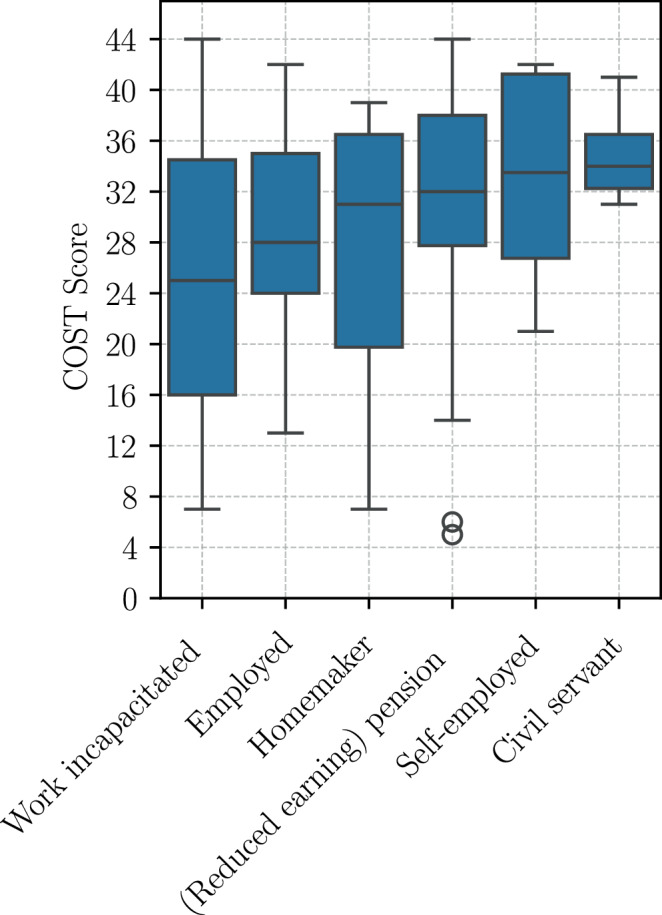
Employment at baseline in relation to COST scores at 3 months after radiation therapy

The most significant predictor of FT at the 3‑month follow-up (Appendix Table 15) was again identified as financial difficulties (*p* = 9.99 $\cdot$ 10^−13^). Additionally, highly significant socioeconomic parameters included net household income (*p* = 0.005) and change in employment situation after radiation (*p* = 0.005). The employment status at baseline (*p* = 0.03), sense of coherence (*p* = 0.035), and QLQ fatigue levels at baseline (*p* = 0.049) were also found to be significant. Work-incapacitated patients (median 25) at baseline had significantly higher FT at 3 months after RT compared to retired patients (median 32), as shown in Fig. 6. The remaining predictor variables, including household size, insurance type, age, gender, system therapy, and tumor stage, did not attain the required statistical significance level.

#### Multivariate regression models with treatment-associated predictors

The following radiation-associated factors were not found to significantly contribute to the formation of FT at any timepoint: tumor stage, health insurance, systemic therapy, hospitalization, change of treatment plan, prior radiation, planned number of fractions, difference between planned and actual number of fractions, gender, and palliative versus curative therapy. The distance to the radiation center was determined in the stepwise selection process because the difference in the AIC was nearly zero at all timepoints.

At baseline (Appendix Table 16), higher age, higher income, and better performance status as measured by the baseline KPS, were significantly associated with higher COST scores, indicating lower FT. Conversely, being diagnosed with pelvic cancer was associated with significantly lower COST scores (β = −6.43, *p* = 0.031), reflecting an increased financial burden. The following predictors were found to be nonsignificant: insurance status, system therapy, hospitalization, change of treatment, prior radiation, differences in radiation fractions, planned radiation fractions, and treatment goal.

After radiation therapy (Appendix Table 17), only higher age (β = 0.18, *p* = 0.005) and higher income (β = 0.002, *p* = 0.005) were significant predictors of higher COST scores. The analysis revealed no statistically significant associations with gender, tumor diagnosis, UICC tumor stage, systemic therapy, insurance type, hospitalization, change in therapy, prior radiation, planned radiation fractions, difference between planned and actual number of fractions, treatment goal, and KPS at baseline.

At 3 months post-RT (Appendix Table 18), higher age (*p* = 0.011) and higher income (*p* = 0.01) continued to predict higher COST scores, i.e., lower FT. Additionally, a diagnosis of head and neck cancer was associated with lower COST scores compared to breast cancer (β = −9.154, *p* = 0.012), indicating higher financial toxicity in this group. No statistically significant differences were observed in baseline KPS, insurance status, systemic therapy, hospitalization, change of treatment, prior radiation, difference in radiation fractions, planned radiation fractions, or treatment goal.

Across all timepoints, income and age consistently predicted FT, highlighting their persistent influence on financial wellbeing throughout treatment. Tumor entity showed significant baseline and 3‑month follow-up differences but did not predict post-RT FT significantly in multivariate models.

#### Model performances

The qq-plots of all models showed that the residual data did not deviate significantly from a normal distribution. Furthermore, the descriptive statistics of the residuals demonstrate symmetry and a median of nearly 0. The scatter plots of residuals and predicted values displayed homoscedasticity, which ensures uniform variance of the residuals across different levels of the independent variables.

The multivariate linear regression models with secondary outcomes demonstrated strong explanatory power across timepoints. At baseline, the pooled model achieved an adjusted R^2^ of 0.565 (CI: 0.456–0.659) and minimal missing information (FMI < 0.006). After RT, the adjusted R^2^ was 0.56 (CI: 0.45–0.66), with an FMI of 0.023. At 3‑month follow-up, model performance remained stable, with an adjusted R^2^ of 0.57 (CI: 0.46–0.66) alongside negligible FMI (< 0.003).

The multivariate linear regression models based on treatment and tumor variables yielded notably lower explanatory power. At baseline, the adjusted R^2^ was 0.26 (CI: 0.15–0.38). The post-RT adjusted R^2^ declined further: 0.19 [CI: 0.09–0.31]. At 3 months, the model yielded an adjusted R^2^ of 0.24 (CI: 0.13–0.36). FMI values ranged from 0.025 to 0.038 across timepoints.

## Discussion

This longitudinal study investigated the course and predictors of financial toxicity in cancer patients undergoing radiation therapy in Germany, a patient population which might be particularly vulnerable. Our findings suggest that FT does not universally increase during or after RT. Rather, the data reveal a slight but consistent decrease in FT across all timepoints, which may reflect specific contextual conditions in the German healthcare system or result from patient-level resilience factors.

Financial toxicity was observed in 40.4% of patients at baseline, decreasing after RT and further until the 3‑month follow-up. This is in line with international literature reporting a wide prevalence range of 16–73% [13], possibly due to differences in healthcare systems, definitions, or instruments used. In the SOCOFIN study, 34.8% of patients experienced at least mild FT at the end of RT, which is consistent with the multicenter cross-sectional study conducted by Fabian et al. in Germany reporting that approximately one third of patients felt burdened by subjective financial distress at the end of radiation treatment [10]. The improving trend in the subgroup of patients experiencing mild FT at baseline should be interpreted cautiously: Although the median COST score was observed to increase until up to 24 points at 3 months after RT, it should be noted that a COST score below 26 still indicates persistent mild FT. This finding suggests that moderate initial financial distress may be amenable to intervention or adaptation during RT.

This prospective single-center clinical trial confirmed several previously described predictors of FT. The most robust predictor throughout all analyses and timepoints was baseline financial difficulties. This aligns with international findings, where financial stress before or at the beginning of cancer treatment has been consistently associated with later development of FT [12]. In the SOCOFIN study, multivariate linear regression models demonstrated highly significant associations between pretreatment financial difficulties and later FT, independent of the tumor diagnosis or clinical parameters. This underscores the importance of early identification of at-risk patients, particularly as subjective financial distress is potentially modifiable. Furthermore, fluctuations in employment status, net income levels, and KPS exhibited a substantial influence on FT, thereby underscoring the multifaceted nature of the economic distress experienced by cancer patients. Better functional status at baseline predicted lower FT at baseline and was correlated with lower FT at 3‑month follow-up (*p* = 0.032).

As observed in earlier studies, patients with lower household income experienced higher levels of FT throughout all regression models in the present study. Furthermore, the SOCOFIN study confirmed the previously identified risk factors of low net income and income loss in predicting financial distress at the end of RT in Germany, as evidenced by multivariate ordinal regression analyses [10, 11]. The results confirm the crucial role of socioeconomic status including employment, income, and level of education, as found by other investigations [4, 12, 16, 17]. In the context of Germany, where out-of-pocket treatment costs are generally lower, the impact of lost income and employment status becomes even more relevant [7, 8, 10, 11].

The deterioration or loss of employment following RT was significantly associated with worse FT at all timepoints in this study. Patients with unchanged employment situations exhibited significantly better COST scores than those whose employment status had worsened. In contrast, the groups with an improved employment situation might have been too small to reach significance after RT (*n* = 3) and 3 months thereafter (*n* = 8). These findings support those of Harris et al. [18], who identified occupational change as a major determinant of FT in palliative RT patients. In the SOCOFIN study, higher income at baseline predicted lower FT at all timepoints in multivariate regression analyses. In this study cohort, stable employment and income were protective, which highlights the importance of occupational support and reintegration efforts during cancer treatment. Moreover, fatigue at baseline was predictive of higher FT at the 3‑month follow-up, which is consistent with the idea that treatment-related symptom burden exacerbates financial vulnerability, particularly when symptoms interfere with work capacity.

Psychosocial parameters also played a clinically relevant role: A higher sense of coherence, which might be concomitant with better coping resources, was significantly associated with lower FT at both baseline and follow-up in this study. Coherently, the number of patients applying coping strategies increased over the course of this study while FT decreased. The increase in applied coping mechanisms, in combination with the overall decrease in FT, may indicate that sufficient coping mechanisms could alleviate FT.

The regression models with treatment-associated parameters identified income level and age to significantly predict FT at all timepoints. The KPS was shown to predict FT at baseline, while specific tumor diagnoses were predictive of FT at baseline and at the 3‑month follow-up. Younger patient age significantly predicted higher FT at all timepoints and was correlated with higher FT after RT as well as at the 3‑month follow-up. The finding that patients with pelvic tumors experienced the highest levels of FT at baseline, while those with head and neck cancers had the highest levels after RT and at the follow-up, is in line with previous observations that these tumor groups are particularly vulnerable [13, 18]. The time-dependent financial burden on different tumor patients underscores the dynamic nature of financial strain across cancer diagnoses. It also highlights potential variations in treatment-associated financial burdens, suggesting that tumor-specific treatment protocols, the profiles of adverse effects, and the associated financial costs may have a unique impact on financial toxicity. Contrary to the findings reported in available literature [5], this study found that breast cancer patients were not subject to a heightened risk of FT. Nonetheless, the tumor diagnosis did not remain a significant independent predictor in multivariate analyses after RT, indicating that socioeconomic variables outweigh tumor type in their explanatory power regarding FT in the SOCOFIN cohort. This hypothesis is supported by the finding that more than every second head and neck cancer patient does not return to work due to disability resulting in lifetime income loss [6]. In the SOCOFIN study, SCCHN patients were distinguished as the sole diagnosis group that exhibited a consistent decline in COST scores, even resulting in lower COST scores at follow-up compared to the baseline measurement.

Interestingly, factors like tumor stage, hospitalization, systemic therapy, or distance to the treatment center did not significantly predict FT, although they have been reported in the international literature as relevant [13, 16, 17]. It is possible that support services, infrastructure, or reimbursement schemes in the German system mitigate their impact to some extent. While systemic therapy was associated with higher FT in univariate tests, it did not remain an independent predictor in the multivariate regression models. This finding indicates that systemic therapy itself was not a primary cause of FT in this study. Rather, it is a concomitant factor that interacts with correlated socioeconomic and psychosocial parameters. The elevated FT observed in patients with pelvic and HNSCC may be, at least in part, attributable to the increased frequency of systemic treatment and its associated consequences.

The divergent performances of the two different regression models lend support to the hypothesis that socioeconomic variables play a more significant role in the development of FT than treatment-associated variables.

### Strengths and limitations

The SOCOFIN study constitutes the first prospective longitudinal study of FT in Germany as well as a comprehensive and exploratory assessment of cancer patients’ wellbeing. The study population is characterized by heterogeneity, comprising a substantial sample of 170 patients with a wide range of tumor diagnoses, ages, tumor stages, and treatment regimens. It is, however, limited by the fact that it is based on data from a single cancer center. Therefore, further multicenter trials are needed to obtain more information about vulnerable subgroups of cancer patients and their characteristics that require specialized support.

## Conclusion

This study shows that financial toxicity in radiation oncology patients may be the most severe even before the initiation of radiation therapy, highlighting the importance of early intervention. Socioeconomic and psychosocial factors—particularly baseline financial difficulties, income, employment stability, and the sense of coherence—were stronger predictors of FT than tumor- or treatment-associated variables. A novel contribution lies in the identification of the sense of coherence as a key resilience factor against worsening FT. These findings support the implementation of structured screening and intervention strategies targeting financial distress, job security, and psychological resilience.

Financial toxicity in this study cohort was shaped rather by subjective burden and systemic vulnerabilities than by direct treatment costs. Future multicenter research should focus on validating these predictors and evaluating interventional models combining financial counseling and psychosocial support to reduce long-term economic consequences for cancer patients.

## Figures and Tables

**Fig. 7:**
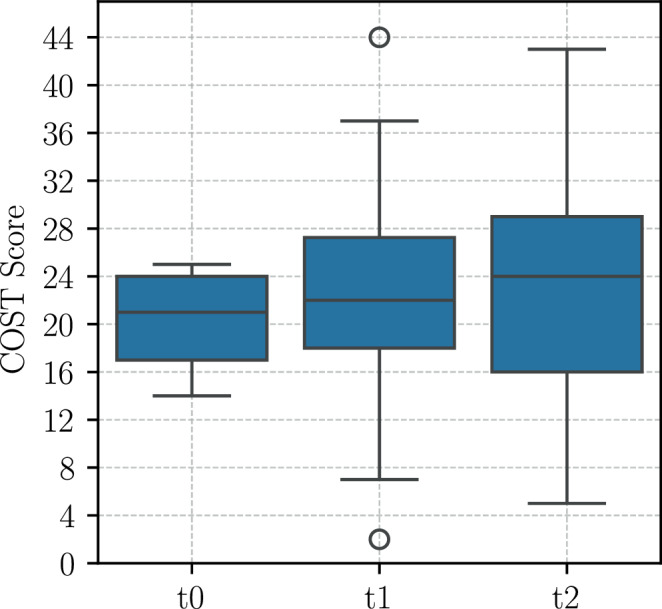
COST scores over time in the subgroup with mild financial toxicity at baseline

**Fig. 8:**
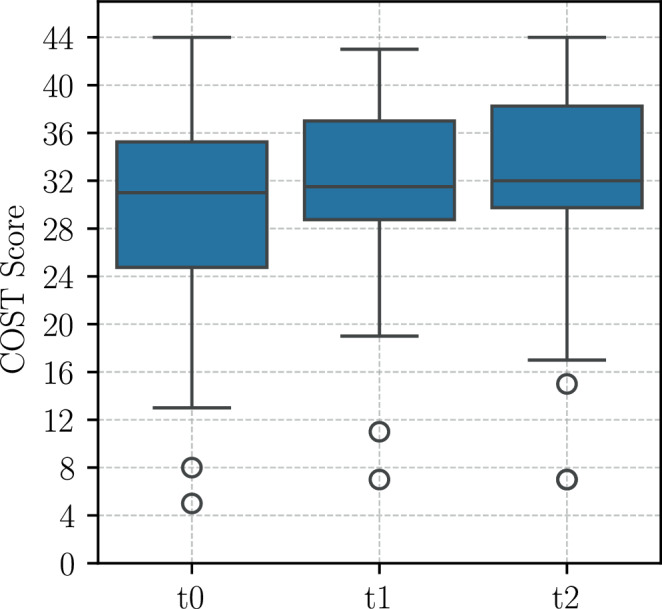
COST scores over time in the subgroup with monthly net income of 2500 to 3500 euros

## Appendix

​

**Table 2:** Severity of financial toxicity over time

	Patient count	Proportion in %
*Before RT*		
G0: no FT	96	59.6
G1: mild FT	56	34.8
G2: moderate FT	9	5.6
*After RT*		
G0: no FT	105	65.2
G1: mild FT	48	29.8
G2: moderate FT	8	5.0
*At 3‑month follow-up*		
G0: no FT	112	69.6
G1: mild FT	42	26.1
G2: moderate FT	7	4.3

**Table 3:** Repeated-measures Friedman test results of the primary outcome over time

	W	ddof1	Q	*p*-unc	Significance
COST score	0.025	2	8.14	0.017	**

*ddof1* delta degrees of freedom with n–1, *p‑unc* uncorrected *p*-value

**Table 4:** Post-hoc Dunn–Bonferroni test results of the primary outcome over time

Comparison	*P*-value	Significance
COST score at baseline vs. COST score after RT	1.00	–
COST score at baseline vs. COST score at 3‑month follow-up	0.29	–
COST score after RT vs. COST score at 3‑month follow-up	0.87	–

**Table 5:** Repeated-measures Friedman tests of the primary outcome over time in subgroups according to FT severity at baseline

FT severity	W	ddof1	Q	*p*-unc	Significance
G0: no FT	0.009	2	1.71	0.43	–
G1: mild FT	0.072	2	8.10	0.02	*
G2: moderate FT	0.309	2	5.55	0.06	–

*ddof1* delta degrees of freedom (n–1), *p‑unc* uncorrected *p*-value

**Table 6:** Post-hoc Dunn–Bonferroni tests of the primary outcome over time in subgroups according to FT severity at baseline

FT severity	Timepoint1	Comparison	Adjusted *p*-value	Significance
G1: mild FT	COST_score_t0_b	COST_score_t1_c	0.4	–
G1: mild FT	COST_score_t0_b	COST_score_t2_3	0.06	–
G1: mild FT	COST_score_t1_c	COST_score_t2_3	1.0	–

**Table 7:** Kruskal–Wallis tests of the primary outcome among subgroups according to tumor diagnosis

Variable	Timepoint	ddof1	H	*p*-unc	Significance
Tumor diagnosis	COST at baseline	6	16.64	0.01	**
Tumor diagnosis	COST after RT	6	15.60	0.02	**
Tumor diagnosis	COST at follow-up	6	17.19	8.59e-03	***

*ddof *delta degrees of freedom (n–1), *p‑unc* uncorrected *p*-value

**Table 8:** Post-hoc Dunn–Bonferroni tests of the primary outcome among subgroups according to tumor diagnosis at baseline (*p*-values)

	Other cancer	Pelvic cancer	Gastrointestinal cancer	Head and neck cancer	Lung cancer	Breast cancer	Prostate cancer
Other cancer	1.00	0.09*	1.00	1.00	1.00	1.00	1.00
Pelvic cancer	0.09*	1.00	1.00	1.00	0.52	0.04**	0.01**
Gastrointestinal cancer	1.00	1.00	1.00	1.00	1.00	1.00	1.00
Head and neck cancer	1.00	1.00	1.00	1.00	1.00	1.00	0.62
Lung cancer	1.00	0.52	1.00	1.00	1.00	1.00	1.00
Breast cancer	1.00	0.04**	1.00	1.00	1.00	1.00	1.00
Prostate cancer	1.00	0.01**	1.00	0.62	1.00	1.00	1.00

**Table 9:** Post-hoc Dunn–Bonferroni tests of the primary outcome among subgroups according to tumor diagnosis 3 months after RT (*p*-values)

	Other cancer	Pelvic cancer	Gastrointestinal cancer	Head and neck cancer	Lung cancer	Breast cancer	Prostate cancer
Other cancer	1.00	0.21	1.00	0.04**	1.00	1.00	1.00
Pelvic cancer	0.21	1.00	1.00	1.00	1.00	0.34	0.22
Gastrointestinal cancer	1.00	1.00	1.00	0.57	1.00	1.00	1.00
Head and neck cancer	0.04**	1.00	0.57	1.00	0.55	0.06*	0.04**
Lung cancer	1.00	1.00	1.00	0.55	1.00	1.00	1.00
Breast cancer	1.00	0.34	1.00	0.06*	1.00	1.00	1.00
Prostate cancer	1.00	0.22	1.00	0.04**	1.00	1.00	1.00

**Table 10:** Mann–Whitney U tests between subgroups of secondary outcomes regarding COST score over time

Mediator	U	Test group	Reference group	*n* (test)	*n* (ref)	*p-*value	Significance
*COST at baseline*							
Hospitalization during RT	2380.5	No hospitalization	Hospitalization	129	32	0.18	–
Hospitalization during 3 months after RT	2375.0	No hospitalization	Hospitalization	130	31	0.12	–
Systemic therapy	3880.5	None or hormonal therapy	Chemotherapy, targeted therapy, immunotherapy	71	90	0.02	*
Gender	2694.0	Female	Male	91	70	0.09	–
*COST after RT*							
Hospitalization during RT	2427.0	No hospitalization	Hospitalization	129	32	0.12	–
Hospitalization during 3 months after RT	2307.0	No hospitalization	Hospitalization	130	31	0.21	–
Systemic therapy	3812.0	None or hormonal therapy	Chemotherapy, targeted therapy, immunotherapy	71	90	0.04	*
Gender	2823.0	Female	Male	91	70	0.22	–
*COST at follow-up*							
Hospitalization during RT	2230.5	No hospitalization	Hospitalization	130	31	0.36	–
Hospitalization during 3 months after RT	2393.0	No hospitalization	Hospitalization	129	32	0.16	–
Systemic therapy	3881.0	None or hormonal therapy	Chemotherapy, targeted therapy, immunotherapy	71	90	0.02	*
Gender	2783.5	Female	Male	91	70	0.17	–

**Table 11:** Spearman correlation Ccoefficients with impact on FT

	COST at baseline	COST after RT	COST at 3‑month follow-up
QLQ financial difficulties at baseline	r = −0.65, *p* = 5.23e-21	r = −0.62, *p* = 1.00e-18	r = −0.65, *p* = 1.05e-20
Monthly net income per household	r = 0.42, *p* = 3.45e-08	r = 0.34, *p* = 7.71e-06	r = 0.35, *p* = 5.12e-06
Change of income after RT	r = 0.41, *p* = 7.27e-08	r = 0.44, *p* = 4.29e-09	r = 0.35, *p* = 4.06e-06
KPS	r = 0.24, *p* = 0.002	Not significant (*p* = 0.086)	r = 0.17, *p* = 0.032
Age	r = 0.24, *p* = 0.002	r = 0.25, *p* = 0.001	r = 0.24, *p* = 0.002
SOC	r = 0.38, *p* = 7.49e-07	r = 0.38, *p* = 6.65e-07	r = 0.35, *p* = 5.19e-06
Fatigue at baseline	r = −0.25, *p* = 0.0016	r = −0.18, *p* = 0.025	r = −0.19, *p* = 0.018
Tumor stage at baseline	r = 0.15, *p* = 0.51	r = 0.22, *p* = 0.004	r = 0.25, *p* = 0.002
Distance to radiation center	Not significant (*p* = 0.375)	Not significant (*p* = 0.375)	Not significant (*p* = 0.904)

**Table 12:** Coping strategies for mitigating financial burden after RT (t1) and 3 months thereafter (t2). Multiple-choice questions; sum may exceed 100%

Coping Strategy	Count t1	Proportion (%) t1	Count t2	Proportion (%) t2
Passive financial resources	38.0	22.4	42.0	24.7
Active financial spending	2.0	1.2	4.0	2.4
Coping with lifestyle	45.0	26.5	59.0	34.7
Support seeking	8.0	4.7	9.0	5.3
Coping with care	4.0	2.4	4.0	2.4
Other	7.0	4.1	7.0	4.1
None of the above	80.0	47.1	80.0	47.1

**Table 13:** Pooled regression results of linear regression models for COST scores at baseline with secondary outcomes

Term	Estimate	SE	t‑value	95% CI lower	95% CI upper	*p*-value	Significance
Intercept	8.38	6.562	1.277	−4.482	21.243	0.204	–
Net income per household	0.001	3.72e-04	3.799	6.84e-04	0.002	2.43e-04	***
Number of people per household	−1.041	0.533	−1.951	−2.087	0.005	0.053	–
QLQ pain at baseline	−0.025	0.02	−1.279	−0.064	0.014	0.203	–
KPS at baseline	0.129	0.056	2.305	0.019	0.239	0.023	*
Unchanged income (vs. decreased) after RT	1.915	1.217	1.573	−0.471	4.301	0.118	–
Increased income (vs. decreased) after RT	8.766	6.216	1.41	−3.416	20.949	0.161	–
No change of occupational situation (vs. deterioration) after RT	1.587	1.455	1.091	−1.265	4.439	0.278	–
Improvement of occupational situation (vs. deterioration) after RT	11.748	3.753	3.131	4.393	19.104	0.002	**
Sense of coherence score at baseline	0.085	0.04	2.146	0.007	0.163	0.034	*
QLQ dyspnea at baseline	0.037	0.019	1.927	−6.26e-04	0.075	0.056	–
QLQ financial difficulties at baseline	−0.136	0.023	−6.019	−0.18	−0.092	1.45e-08	***
Employment at baseline	−1.831	1.126	−1.626	−4.038	0.376	0.106	–
Public insurance (vs. private)	−1.545	1.438	−1.075	−4.363	1.273	0.284	–
Physical work no or partly (vs. yes)	1.549	1.387	1.117	−1.17	4.268	0.266	–
Social network index at baseline	0.459	0.411	1.116	−0.347	1.265	0.266	–

**Table 14:** Pooled regression results of linear regression models for COST scores after RT with secondary outcomes

Term	Estimate	SE	t‑value	95% CI Lower	95% CI Upper	*p*-value	Significance
Intercept	19.84	3.869	5.128	12.257	27.423	9.51e-07	***
Net income per household	7.18e-04	3.87e-04	1.856	−4.04e-05	0.001	0.066	–
Number of people in household	0.893	0.673	1.327	−0.426	2.211	0.187	–
QLQ fatigue at baseline	0.019	0.021	0.905	−0.023	0.061	0.367	–
Sense of coherence score at baseline	0.075	0.04	1.893	−0.003	0.152	0.06	–
Gender (male vs. female)	−1.847	1.062	−1.739	−3.929	0.235	0.084	–
Public insurance (vs. private)	−1.409	1.486	−0.949	−4.321	1.503	0.345	–
Unchanged income (vs. decreased) after RT	2.387	1.188	2.008	0.058	4.716	0.047	*
Increased income (vs. decreased) after RT	8.529	6.268	1.361	−3.756	20.813	0.176	–
No change of occupational situation (vs. deterioration) after RT	4.209	1.413	2.979	1.44	6.978	0.004	**
Improvement of occupational situation (vs. deterioration) after RT	9.578	3.704	2.586	2.318	16.838	0.011	*
QLQ financial difficulties at baseline	−0.16	0.022	−7.16	−0.204	−0.116	4.19e-11	***
Employment at baseline	−2.045	1.104	−1.853	−4.209	0.119	0.066	–
UICC tumor stage I or II (vs. III or IV)	1.345	1.092	1.232	−0.795	3.485	0.22	–
UICC tumor stage not applicable (vs. UICC III or IV)	2.571	1.448	1.775	−0.267	5.41	0.078	–
QLQ nausea/vomiting at baseline	0.03	0.03	0.979	−0.03	0.089	0.329	–
Nonacademic education (vs. no training)	−0.695	1.633	−0.426	−3.895	2.505	0.671	–
Academic education (vs. no training)	1.397	1.969	0.709	−2.463	5.257	0.479	–

**Table 15:** Pooled regression results of linear regression models for COST scores 3 months after RT with secondary outcomes

Term	Estimate	SE	t‑value	95% CI Lower	95% CI Upper	*p*-value	Significance
Intercept	26.698	5.429	4.917	16.057	37.339	2.43e-06	***
Net income per household	0.001	4.30e-04	2.862	3.88e-04	0.002	0.005	**
Number of people per household	−0.711	0.594	−1.198	−1.875	0.453	0.233	–
QLQ fatigue at baseline	0.043	0.021	1.987	5.89e-04	0.085	0.049	*
Sense of coherence score at baseline	0.092	0.043	2.134	0.007	0.176	0.035	*
UICC tumor stage I or II (vs. III or IV)	1.959	1.231	1.592	−0.453	4.372	0.114	–
UICC tumor stage not applicable (vs. UICC III or IV)	2.969	1.587	1.871	−0.141	6.079	0.063	–
Public insurance (vs. private)	−2.66	1.741	−1.527	−6.073	0.753	0.129	–
No change of occupational situation (vs. deterioration) after RT	3.835	1.35	2.841	1.19	6.48	0.005	**
Improvement of occupational situation (vs. deterioration) after RT	5.905	3.927	1.504	−1.791	13.601	0.135	–
QLQ financial difficulties at baseline	−0.186	0.024	−7.877	−0.233	−0.14	9.99e-13	***
Age	−0.112	0.063	−1.784	−0.234	0.011	0.077	–
Gender (male vs. female)	−1.871	1.133	−1.651	−4.091	0.35	0.101	–
No systemic therapy or hormone therapy (vs. systemic therapy)	1.867	1.234	1.513	−0.552	4.285	0.133	–
Retirement (vs. work incapacitation) at baseline	4.066	1.853	2.194	0.434	7.697	0.03	*
Housemaker (vs. work incapacitation) at baseline	1.317	2.822	0.466	−4.215	6.848	0.642	–
Employment (vs. work incapacitation) at baseline	−0.08	1.764	−0.045	−3.536	3.377	0.964	–
Self-employment (vs. work incapacitation) at baseline	3.573	2.551	1.401	−1.427	8.572	0.164	–
Civil service (vs. work incapacitation) at baseline	0.155	3.33	0.047	−6.372	6.682	0.963	–

**Table 16:** Pooled regression results of linear regression models for COST scores at baseline with treatment-associated parameters

Term	Estimate	SE	t‑value	95% CI Lower	95% CI Upper	*p*-value	Significance
Intercept	−8.91	9.598	−0.928	−27.722	9.901	0.355	–
Age	0.197	0.062	3.203	0.077	0.318	0.002	**
Gender (male vs. female)	2.582	1.859	1.389	−1.063	6.226	0.167	–
Prostate cancer (vs. breast cancer)	−3.329	3.138	−1.061	−9.479	2.822	0.291	–
Gastrointestinal cancer (vs. breast cancer)	−3.228	2.637	−1.224	−8.398	1.941	0.223	–
Lung cancer (vs. breast cancer)	0.254	2.767	0.092	−5.17	5.678	0.927	–
Pelvic cancer (vs. breast cancer)	−6.43	2.957	−2.174	−12.227	−0.634	0.031	*
Other/brain tumor (vs. breast cancer)	1.23	3.032	0.406	−4.713	7.172	0.686	–
Head and neck cancer (vs. breast cancer)	−5.977	3.366	−1.776	−12.575	0.621	0.078	–
UICC tumor stage I/II (vs. III/IV)	−0.848	1.746	−0.486	−4.271	2.575	0.628	–
UICC tumor stage not applicable (vs. III/IV)	−1.61	2.832	−0.569	−7.16	3.94	0.57	–
Public insurance (vs. private)	−2.858	1.946	−1.469	−6.673	0.956	0.144	–
No systemic therapy or hormone therapy (vs. systemic therapy)	−0.737	1.719	−0.429	−4.107	2.633	0.669	–
Hospitalization during RT (yes vs. no)	−0.386	1.684	−0.229	−3.688	2.915	0.819	–
No change in tumor therapy (vs. change)	1.635	1.676	0.976	−1.649	4.919	0.331	–
Prior radiation therapy	1.869	3.479	0.537	−4.949	8.687	0.592	–
Difference in planned vs. completed fractions	−0.189	0.158	−1.202	−0.498	0.119	0.231	–
Planned number of radiation fractions	0.032	0.1	0.321	−0.165	0.229	0.749	–
Curative treatment goal (vs. palliative)	−0.244	2.034	−0.12	−4.23	3.742	0.905	–
Net income per household	0.002	5.23e-04	3.257	6.78e-04	0.003	0.002	**
KPS at baseline	0.251	0.078	3.231	0.099	0.404	0.002	**

**Table 17:** Pooled regression results of linear regression models for COST scores after RT with treatment-associated parameters

Term	Estimate	SE	t‑value	95% CI Lower	95% CI Upper	*p*-value	Significance
Intercept	1.819	9.92	0.183	−17.624	21.261	0.855	–
Age	0.18	0.064	2.829	0.055	0.305	0.005	**
Gender (male vs. female)	1.01	1.923	0.525	−2.759	4.779	0.6	–
Prostate cancer (vs. breast cancer)	−2.679	3.25	−0.824	−9.049	3.691	0.411	–
Gastrointestinal cancer (vs. breast cancer)	−0.58	2.731	−0.212	−5.934	4.773	0.832	–
Lung cancer (vs. breast cancer)	−2.277	2.861	−0.796	−7.885	3.33	0.427	–
Pelvic cancer (gynecological/urological) (vs. breast cancer)	−2.388	3.054	−0.782	−8.374	3.597	0.436	–
Other cancer/primary brain tumor (vs. breast cancer)	4.19	3.139	1.335	−1.962	10.343	0.184	–
Head and neck cancer (vs. breast cancer)	−6.325	3.485	−1.815	−13.156	0.507	0.072	–
UICC tumor stage I or II (vs. III or IV)	0.755	1.805	0.418	−2.784	4.293	0.677	–
UICC tumor stage not applicable (vs. UICC III or IV)	−0.908	2.936	−0.309	−6.662	4.847	0.758	–
Public insurance (vs. private)	−2.662	2.005	−1.328	−6.592	1.268	0.187	–
No systemic therapy or hormone therapy (vs. systemic therapy)	0.321	1.778	0.181	−3.164	3.806	0.857	–
Hospitalization during RT	0.487	1.746	0.279	−2.935	3.909	0.781	–
Change in tumor therapy during RT	2.173	1.733	1.254	−1.224	5.57	0.212	–
Previous radiotherapy	2.214	3.597	0.615	−4.837	9.265	0.539	–
Difference of planned to actual fractions	−0.053	0.163	−0.322	−0.372	0.267	0.748	–
Planned radiation fractions	0.056	0.104	0.539	−0.148	0.26	0.591	–
Curative treatment goal	−0.437	2.112	−0.207	−4.577	3.704	0.837	–
Net income per household	0.002	5.19e-04	2.911	4.94e-04	0.003	0.005	**
KPS at baseline	0.121	0.081	1.504	−0.037	0.279	0.135	–

**Table 18:** Pooled regression results of linear regression models for COST scores 3 months after RT with treatment-associated parameters

Term	Estimate	SE	t‑value	95% CI Lower	95% CI Upper	*p*-value	Significance
Intercept	−0.55	10.307	−0.053	−20.751	19.652	0.958	–
Age	0.169	0.066	2.565	0.04	0.299	0.011	*
Gender	1.912	2	0.956	−2.009	5.832	0.341	–
Prostate cancer (vs. breast cancer)	−4.344	3.362	−1.292	−10.933	2.245	0.198	–
Gastrointestinal cancer (vs. breast cancer)	−1.015	2.824	−0.359	−6.549	4.52	0.72	–
Lung cancer (vs. breast cancer)	1.569	2.96	0.53	−4.231	7.37	0.597	–
Pelvic cancer (gynecological/urological) (vs. breast cancer)	−3.458	3.19	−1.084	−9.711	2.794	0.28	–
Other cancer/primary brain tumor (vs. breast cancer)	3.202	3.247	0.986	−3.163	9.566	0.326	–
Head and neck cancer (vs. breast cancer)	−9.154	3.609	−2.536	−16.227	−2.08	0.012	*
UICC tumor stage I or II (vs. III or IV)	1.488	1.875	0.794	−2.187	5.163	0.429	–
UICC tumor stage not applicable (vs. UICC III or IV)	−0.341	3.036	−0.112	−6.292	5.61	0.911	–
Public insurance (vs. private)	−3.15	2.077	−1.516	−7.221	0.922	0.132	–
No systemic therapy or hormone therapy (vs. systemic therapy)	0.883	1.839	0.48	−2.722	4.488	0.632	–
Hospitalization during RT	−0.072	1.818	−0.04	−3.635	3.491	0.968	–
Change in tumor therapy during RT	2.834	1.913	1.482	−0.915	6.582	0.143	–
Previous radiotherapy	1.688	3.757	0.449	−5.676	9.051	0.654	–
Difference of planned to actual fractions	−0.165	0.169	−0.976	−0.495	0.166	0.331	–
Planned radiation fractions	0.101	0.107	0.942	−0.109	0.312	0.348	–
Curative therapy (vs. palliative)	0.115	2.191	0.053	−4.179	4.409	0.958	–
Net income per household	0.001	5.56e-04	2.644	3.80e-04	0.003	0.01	*
KPS at baseline	0.149	0.083	1.79	−0.014	0.312	0.076	–
